# Sensory quality of soymilk and tofu from soybeans lacking lipoxygenases

**DOI:** 10.1002/fsn3.274

**Published:** 2015-08-26

**Authors:** Aijun Yang, Heather Smyth, Mridusmita Chaliha, Andrew James

**Affiliations:** ^1^ CSIRO Agriculture306 Carmody RoadSt LuciaQueensland4067Australia; ^2^ Queensland Alliance for Agriculture and Food InnovationThe University of QueenslandSt LuciaQueensland4072Australia; ^3^ Department of Agriculture, Fisheries and ForestryCoopers PlainsQueensland4108Australia

**Keywords:** Lipoxygenase, sensory quality, soy milk, soybean, tofu

## Abstract

The oxidation of unsaturated lipids by lipoxygenases in soybeans causes undesirable flavors in soy foods. Using a traditional and a nontraditional soy food user group, we examined the cultural difference in perceiving the sensory characteristics of soymilk and tofu produced from soybeans with or without lipoxygenases (Lx123). The two groups described the samples using similar terms. The traditional users preferred the control soy milk and lipoxygenase‐free tofu while the nontraditional users preferred the lipoxygenase‐free soymilk with no preference for tofu. In a separate study, a trained descriptive taste panel compared the odor of soymilk and tofu from control soybeans or those lacking lipoxygenase‐1 and lipoxygenase‐2 (Lx12) or all three isomers (Lx123). The rancid/grassy odor was rated the lowest in Lx123 products, followed by Lx12 products with the control products given the highest rating. The Lx12 and Lx123 products were also sweeter and less bitter than the controls. Taken together, our results demonstrated that soybeans lacking lipoxygenases can produce soy foods with less undesirable aromas and are therefore likely more acceptable to the consumers.

## Introduction

Soy foods produced from whole soybean seeds such as soymilk and tofu have been part of the traditional foods in East Asia for a long time. Soybean is also an important ingredient in many processed foods. Its production and use has increased rapidly due to its high nutritional value and potential health benefits. Utilization of soybean seeds as food materials, however, has sometimes been limited, particularly in Western societies, because of their “grassy/beany” flavor (Krishna et al. 2003) and certain consumers prefer a bland or neutral flavor in soy products. The consumers from traditional soy food consumption countries, on the other hand, generally favorably associate beany flavor with soy products such as soymilk and tofu. Cross‐cultural variations in food preferences are well known and cultural differences in consumption of soy products might account for some differences in perception of sensory attributes. These undesirable flavors, characterized as beany, green, grassy, painty, astringent, and bitter, have been associated with off‐flavors from the oxidation of polyunsaturated lipids by lipoxygenases (LOX) present in soybeans.

Soybeans are known to be a rich source of LOX and mature soybean seeds contain three major lipoxygenases: LOX‐1 (lipoxygenase‐1), LOX‐2 (lipoxygenase‐2), and LOX‐3 (lipoxygenase‐3). Soybean also contains high amount of polyunsaturated fatty acids (PUFA), predominantly linoleic acid (C18:2) at around 50% and linolenic acid (C18:3) up to 11% (King et al. 1998; Gerde and White 2008). Both C18:2 and C18:3 contain a *cis,cis*‐1,4‐pentadiene structure potentially leading to the production of hydroperoxides when oxidized, which in turn are converted into volatile compounds associated with undesirable flavors (Kitamura 1984). Full‐fat soy flour is especially prone to such deterioration and has a disagreeable taste that is difficult to mask. Before beans are crushed or ground, LOX and PUFA are separated within the cell, but following soaking and homogenization in the process of making soymilk, they are mixed and begin to react to form the oxidation products and volatile compounds. Among the volatile compounds detectable in soy products, hexanal is primarily responsible for the objectionable flavor or aroma of soy foods and has a very low detection threshold (Wilkens and Lin 1970). The removal of all or some of the LOX present in soybeans through breeding has produced soybean lines lacking LOX and subsequent lower levels of volatiles responsible for the rancid flavor, which could be useful in improving the acceptance of Western consumers. While LOX‐free soybean has been shown to produce lower hydroperoxide and hexanal levels than that with LOX or lacking LOX‐1, ‐2, or ‐3 isozyme (Hildebrand et al. 1991; Kobayashi et al. 1995; Furuta et al. 1996), there have also been mixed results in the literature on the impact of these LOX‐free soybean lines on controlling the beany flavor of soy foods produced. For example, using GC‐MS, Kobayashi et al. (1995) showed that almost all volatiles in soymilk produced from mutants lacking LOX were markedly lower than those from a normal variety. Wilson (1996) reported that tofu made from LOX‐2 null lines of soybeans were less beany than their respective controls as evaluated by a sensory panel. However, while LOX‐free soybeans reduced the beany flavor in certain types of soy products such as soymilk and tofu (Wilson 1996; Torres‐Penaranda et al. 1998), they showed no effect in oils, breads, and meat patties (King et al. 1998, 2001; Liu et al. 2008). Besides improved flavor and aroma, soybeans null in LOX could be more resistant to adverse storage conditions than the normal soybeans with regard to changes in pH and solid content of the soymilk (Lambrecht et al. 1996), although Torres‐Penaranda and Reitmeier (2001) reported significant sensory differences between soymilks from soybeans lacking LOX stored for 3 months and 15 months and no difference for the soymilks from the normal soybeans stored for these times.

The LOX‐null lines, including those developed in Australia, have been shown to have similar agronomic traits including yield (Narvel et al. 1998; Reinprecht et al. 2006) (A. James, 2013 unpubl. data). However, the LOX‐free lines from Australia have not been evaluated for their effect on storage stability, beany flavor, and other quality attributes in soy foods. There are two types of commercial soymilk in Australia, one made from imported soy protein isolates and one made from domestically sourced whole soybean. The grassy/beany flavor of the whole bean soymilk is reported to be a limitation on the uptake of this type by the market. Therefore, soybean without lipoxygenase may offer an opportunity to expand the market for Australian‐grown soybeans. Studies using panels from different cultural backgrounds to evaluate the sample product may give some indication as to whether such differences should be considered. The Australian market for soy products has consumers of many cultural and ethnic backgrounds. The objectives of this study were therefore to compare soymilk and silken tofu from normal and lipoxygenase‐free soybeans using a predominantly native Chinese panel (“traditional soy users”) and an Anglo‐Australian panel (“nontraditional soy users”) in Australia and to investigate the impact of LOX‐free soybeans developed in Australia on sensory attributes of soy products in order to improve our understanding of the potential utility of these traits for the Australian market.

## Materials and Methods

### Soybeans, soymilk and tofu

Normal soybeans and soybean lines selected for LOX‐null in Australia were used in these studies. All the lines used were grown, harvested, and stored together before use. In the preliminary study, a commercial line and a genotype lacking all three major LOX isozymes (Lx123) were used. In the following sensory study, normal soybeans and lines lacking LOX‐1 and LOX‐2 (Lx12) or all three isomers (Lx123) were selected. The LOX‐1 and LOX‐2 genes are tightly linked and inherited together, while the LOX‐3 gene is independently inherited (Davies and Nielsen 1986). All the materials were kept at around 22°C until use.

**Figure 1:**
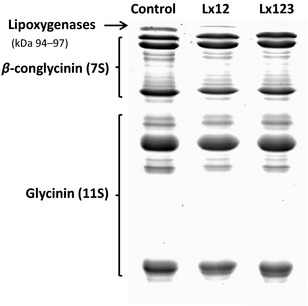
Sodium dodecyl‐polyacrylamide gel electrophoresis profile of seed proteins from control, Lx12 or Lx 123 soybeans.

All soybean lines were checked for LOX using a screening method (Suda et al. 1995; Narvel et al. 2000) and SDS‐PAGE (sodium dodecyl‐polyacrylamide gel electrophoresis) (Yang and James 2013) to confirm the absence of LOX‐1 and LOX‐2 or all three LOX isomers. A typical SDS‐PAGE profile of soybean seed proteins with or without LOX isozymes is presented in Figure 1.

Soymilk and silken tofu were produced according to the procedure optimized by Yang and James (2013) and stored at 4°C overnight before use in sensory and focus group assessments. Briefly, the beans were soaked overnight before they were ground in a domestic blender. The slurry was cooked at 98°C and then filtered using a juice extractor to obtain soymilk. The required amount of the soymilk for assessment was transferred to 4°C. The remaining soymilk was made into silken tofu by adding 0.3% coagulant (nigari, mostly magnesium chloride) and allowing the curd to form at 85°C. The soymilk and tofu were kept at 4°C until use.

### Preliminary study of soymilk and tofu from control and LOX‐free soybeans

#### Sensory evaluation using panels of “traditional” and “nontraditional” soy users

Two semiformal focus groups were held (each of 1 h duration) involving two target groups namely “nontraditional soy users” (the Australian panel) and “traditional soy users” (the Chinese panel). The profiles of the participants for each group are given in Table 1. The participants in each group were presented samples of soy milk and silken tofu made from commercial beans (control) and lipoxygenase‐free soybeans (LOX‐free) together with paper questionnaires. Participants were asked to describe the appearance, aroma, texture and mouth feel, flavor, and aftertaste of samples and to rate the overall acceptability of each sample on an unstructured 15‐cm hedonic line scale anchored from “dislike extremely” to “like extremely”. The individual assessments were made under controlled conditions (light, temperature) and samples were labeled with 3‐digit codes. Presentation order of samples was identical across the participants as follows: control soy milk, LOX‐free soy milk, control silken tofu, and LOX‐free tofu. Samples (15–20 g each) were presented in disposable plastic pots (tofu) and cups (milks). Participants were also asked to complete a short demographic questionnaire and indicate their soy product knowledge and consumption behavior. Following the taste session, a focus group discussion was held in which participants were asked to share and discuss their thoughts on the sensory properties and acceptability of the samples and their past soy product experiences and consumption behavior.

**Table 1:** Profiles of participants involved in soy focus group study

		Age range (years)	Ethnicity	Soy product consumption
Nontraditional soy product users	2 male5 female	20–70	Anglo‐Australian	Daily – never
Traditional soy product users	1 male5 female	40	Chinese	Daily – weekly

#### Instrumental analysis of volatile compounds by GC‐MS

Replicated samples of soy milk and silken tofu (control and LOX‐free) after 1 day and 8 days stored at 4°C were sampled and frozen (−19°C) for volatile analysis. Aliquots of 5 mL of soy milk or 5 g of silken tofu were placed in 25 mL glass vials for SPME (solid phase microextraction) and immediately closed with screw caps with PTFE/silicone septa (Supelco, Bellefonte, PA).

The method used for volatile analysis was a modified version of the method reported by Achouri et al. (2006). Samples were analyzed with a 6890N GC (gas chromatograph) equipped with a 5975 MSD (mass spectrometric detector) (Agilent Technologies, Palo Alto, CA). The GC was fitted with a DB‐WAX column (J&W Science, i.d. = 0.25 *μ*m, length = 30.0 m, film thickness = 0.25 *μ*m), and helium (BOC gases, ultrahigh purity) was used as a carrier gas at a linear velocity of 56 cm/min and at a flow rate of 2.4 mL/min.

The polydivinylbenzene and carbowax and polydimethylsiloxane (DVB–CAR–PDMS, Gray, 50/30 *μ*m, Supelco) fiber was introduced into the headspace of the sample vial and was allowed to incubate at 45°C for 20 min. After the incubation, the fiber was retracted into a needle and desorbed into the injection port of the GC for 6 min. The fiber remained in the injection port for 6 min to eliminate the possible residues on the fiber. Extraction was supported by magnetic stirring at 250 rpm/s and a fiber blank experiment was performed. The mass spectrometer was operated in the electron impact ion mode with a source temperature of 250°C. The electron energy was 70 eV and the mass range was 35–300 *m/z*. The GC oven temperature started at 35°C for 3 min, was increased at 6.0°C/min to 220°C, and was held for 10 min.

Data analysis was carried out with MSD ChemStation Data Analysis software (Agilent Technologies) to obtain peak responses of volatiles. Peak identification was achieved by comparison of spectra and retention times with authentic reference standards.

### Sensory study on comparing control, Lx12, and Lx123 soy products

#### Soybean seed characterization

The moisture, oil, and protein content of the soybeans were determined as described by Yang and James (2013). The seeds were analyzed for their fatty acid composition using GC. Briefly, soybean seeds were ground to a fine powder and oil was extracted twice with n‐hexane. The combined hexane extractions were evaporated under nitrogen. The oil, 0.1 g, was methylated with 0.5 mol/L sodium methoxide solution at 80°C for 10 min. After cooling to room temperature, 0.05 mL of glacial acetic acid, 2.5 mL of distilled water, and 2.5 mL of petroleum spirit (or hexane) were added. The mixture was vortexed and 0.5 *μ*L of the clear upper phase was used for analysis. The separation and distribution of fatty acid methyl esters were obtained using GC (Perkin Elmer Autosystem Gas Chromatograph, MA, USA) fitted with a FID (flame ionization detector) and a capillary column (SGE BPX70, 25 m × 0.22 mm). The carrier gas was helium at 15 psi. The injector temperature was 240°C and detector temperature was 280°C. The oven temperature started at 150°C before reaching 200°C.

The ground full‐fat soybean meal was also stored at 37°C for 1 week and the total volatiles were measured using a SPME‐GC‐FID procedure.

#### Sensory evaluation

Three replicates of each silken tofu and soy milk sample were prepared from the composite samples of soybeans the day prior to the commencement of sensory testing and training and formal assessments were completed within a 3 day period to ensure freshness of samples. Sensory descriptive analysis techniques were applied to assess and quantify the major sensory properties of six samples including tofu and soy milk made from commercial soybeans (control), from triple‐null soybeans (Lx123), and from double‐null soybeans (Lx12). A total of 11 panelists (four male, seven female), who were experienced in descriptive sensory studies and who were staff and students at the Health and Food Sciences Precinct, Coopers Plains, Queensland, participated in the study. Their profiles are listed in Table 1.

Two training sessions were conducted in a board‐room style round table suitable for discussions. The first training session involved presentation of all samples to panelists, individual aroma and taste assessments, and vocabulary development through discussion (Table 2). The second training session involved practicing rating attributes for each of the samples and developing a concise list of attributes, scales, and definitions. Attributes selected during training, together with their definitions, are shown in Table 3.

**Table 2:** Sensory descriptors and vocabulary from the training session in the preliminary evaluation of control and LOX‐free soymilk and tofu

LOX‐free soymilk	Control soymilk
Yellowish Cooked pasta, floury, evaporated milk, cooked, sour, grassy, raw, less intense/subtle, beany, sweet floral, raw soySoy, painty, potato, alfalfa sprouts, snow pea, green‐sweet, macadamia, savory, soft‐nutty sourness, sweeter, less intense	Off‐white, lighter color, thicker looking“Typical” Chinese‐style, strong, stale‐earthy, starchy, raw beans, green, floral, nutty, chalky, rancid, flour and water paste, raw green peaRaw beans, soy, fresh veg, grassy, aged oil/rancid, painty, green astringent, earthy, bitterness, legume, dry milk, more intense

LOX‐free tofu	Control tofu
Lighter, yellower More subtle, less intense, green bean, fresh bean, grassy, green‐floral, rubber tree, sappy, fermented, legume, pleasantBitter, metallic, nutty, sourness, potato, dairy, buttery, raw bean, sweeter, fresher, clean aftertaste	Creamy, beigeMore intense, rancid, raw bean, starch, nutty, grain, painty, old rancid wheat bran, flouryRancid, aged oil, sour, low‐intensity flavors, bitterness, raw taste, metallic, green, legume, painty, oxidized, potato

**Table 3:** Sensory attributes and definitions developed and used in the sensory descriptive study of control and LOX‐free soymilk and tofu

Attribute	Definition
*Aroma intensity*	The overall intensity of the samples aroma from subtle to intense
*Fresh pea aroma*	A fresh pea, cucumber, melon‐like or lettuce aroma
*Grassy rancid aroma*	A grassy rancid aroma, sappy, sour, raw and painty, like rancid oil
*Floury aroma*	A floury, starchy, potato‐like aroma, reminiscent of clay and earth
*Flavor intensity*	The overall flavor intensity of the sample when in the mouth
*Sweetness*	The perceived sweetness of samples experienced in the mouth
*Bitterness*	The perceived bitterness of samples experienced in the mouth

Formal assessments were conducted in the sensory laboratory of the Health and Food Sciences Precinct, Coopers Plains, Queensland. The laboratory consists of individual tasting booths equipped with computers, daylight equivalent lighting, and temperature control. Three replicate sessions were held whereby all six samples were presented and assessed according to a balanced presentation design. Data were collected using sensory software Compusense five (version 5.0, Compusense Inc., Guelph, ON, Canada) and data were exported and analyzed using XLSTAT (version 2014.6.05, Addinsoft 1995–2014, CA, USA). During training and formal sessions, samples of tofu and milk (~10–15 g) were presented to panelists in small plastic cups covered with plastic lids. Milks were freshly stirred and tofu was freshly sliced prior to serving.

### Statistical analysis

All measurements in this study were replicated three times. The data were subject to analysis of variance to examine the effect of genotypes on each of the sensory attributes examined in soymilk and tofu. The means were separated using least square difference at the 5% significance level.

## Results

### Preliminary study using panels of “traditional” and “nontraditional” soy users

**Figure 2:**
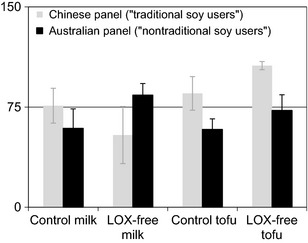
Difference in preference between an Australian (*n* = 8) and a native Chinese (*n* = 6) panel for control and LOX‐free soymilk and tofu.

Typically, the participants from each target group described the samples using similar terms. The control soy milk was paler in color than the LOX‐free soy milk. Both groups described the LOX‐free milk as being less intense and more subtle with more floury, cooked pasta‐type aromas which also came through on the palate together with some sweet green snow pea‐like flavors. The LOX‐free milk was also described as sweeter than the control and less intense in flavor overall. The control soy milk was stronger in odor with raw green pea/bean, stale‐earthy, and rancid‐type aromas. The flavor was also stronger, raw, and grassy with some rancid oil, painty, and bitter‐type flavors. Similarly for the silken tofu samples, the control was described overall as being more intense in odor and flavor. The LOX‐free tofu was described as having fresher/cleaner, more subtle attributes, while the control was described as being rancid (not fresh) and more intense aroma and flavor. Interestingly, the Chinese panel (“traditional soy users”) described the control soy milk as “typical” Chinese‐style whereas they described the LOX‐free milk as unusual. Overall, the Chinese panel preferred the control soymilk and the LOX‐free tofu while the Australian panel (“nontraditional soy users”) preferred the LOX‐free soymilk and did not seem to like either of the tofu products (Fig. 2).

**Figure 3:**
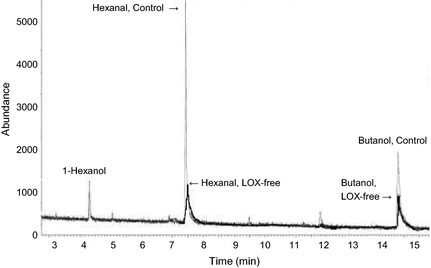
Major volatiles in control soymilk and LOX‐free soymilk.

**Figure 4:**
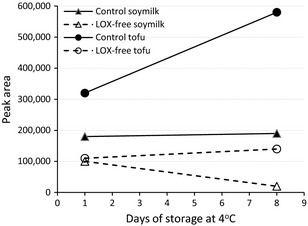
Hexanal peak area for tofu and soymilk after 1 and 8 days of storage at 4°C

The presence or absence of the three major lipoxygenases was confirmed using SDS‐PAGE (Fig. 1) and a screening method. The soymilk from the control and LOX‐free soybean had similar solid content in soymilk (~11%). The major volatiles in these products as determined by GC‐MS were hexanal with some 1‐hexanol and butanol. A GC‐MS profile showing the difference in the major volatiles in soymilk stored at 4°C for 1 day is presented in Figure 3. The peak area data (×100,000) revealed that all three volatiles in the control products were much higher than that in the LOX‐free products, 1.85, 2.75, and 1.22 for control soymilk and 0.60, 2.05, and 0.42 for LOX‐free soymilk for hexanal, 1‐hexanol, and butanol, respectively; 4.5, 2.15, and 1.03 for control tofu and 1.25, 1.75, and 0.40 for LOX‐free tofu for hexanal, 1‐hexanol, and butanol, respectively. The difference in the major volatile, hexanal, between control and LOX‐free products seemed to have increased after 8 days of refrigerated storage (Fig. 4).

### Sensory study on comparing control and Lx12 and Lx123 soy products

The seed characteristics of the soybeans containing the three major lipoxygenases, lacking LOX‐1 and LOX‐2 (Lx12) or lacking all three isomers (Lx123) are presented in Table 4. The similar genetic background of the three soybean types, except for lipoxygenases, was expected to yield products with similar characteristics other than the beany aroma/flavor. While the seeds of Lx12 were smaller and, compared with the controls, contained more protein, the three groups had similar oil content and fatty acids profiles, in particular total saturated fatty acids, total PUFA, and the ratio of linoleic to linolenic fatty acids. As rancidity results from the oxidation of PUFA, the similar fatty acid composition provided an important basis for comparing differences in the sensory attributes or storage stability. After 1 week's storage at 37°C, the total peak area for the major volatiles detected using SPME‐GC‐FID increased over fourfold for the control full‐fat soybean meal while the increase for double‐null and triple‐null samples was only 1.3‐fold (*P *<* *0.05).

**Table 4:** Seed characteristics of control, Lx1‐2 null and Lx1‐3 null samples

	Control	Lx12	Lx123
Seed wt (g/100 seeds)	26.49^a^ a	21.49^b^	25.34^a^
Oil (%)	19.80	20.09	19.75
Protein (%)	39.56^b^	44.33^a^	42.86^ab^
Major fatty acids			
C16:0	10.18	9.99	10.33
C18:0	1.53	1.78	1.63
C18:1c	20.03	20.67	23.36
C18:2	53.34	51.50	49.55
C18:3	9.09	9.52	8.82
PUFA ratio (18:2/18:3) (PR)	5.73	5.41	5.62
Total SFA	14.13	14.25	14.26
Total PUFA	61.53	61.02	58.37
Total PUFA/Total SFA (PI)	4.36	4.28	4.09
Total volatile increase in full‐fat soy meal after 1 week storage at 37°C	4.10^a^	1.32^b^	1.38^b^

SFA, saturated fatty acids; PUFA, polyunsaturated fatty acids; PR, polyunsaturation ratio; PI, polyunsaturation index.

a Means with the same superscripts within the same row are not statistically different, *P *>* *0.05.

**Figure 5:**
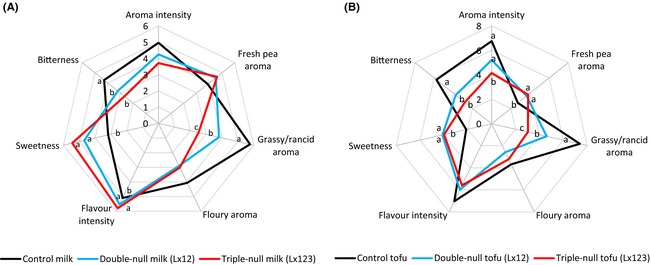
Plot of sensory scores for soymilk (A) and silken tofu (B) samples. *n* = 3 × 11, for each sensory attribute, the scores with a different letter are significantly different (*P* < 0.05).

The sensory analysis of the soy products made from control soybeans or those lacking LOX‐1 and LOX‐2 (Lx12) or all three isomers (Lx123) showed clear and significant differences in the major aroma notes between these three groups for soymilk (Fig. 5A) or silken tofu (Fig. 5B). The objectionable rancid/grassy odors were rated the lowest by the taste panel in both soymilk and tofu from Lx123 soybeans, followed by Lx12 products with the control products given the highest rancid odor rating. The difference between these three soybeans was statistically significant for both soymilk and tofu (*P *<* *0.05). Bitterness, another negative sensory note, was rated significantly lower in the soymilk and tofu made from the soybeans lacking two or three lipoxygenases compared with those made from the soybean containing all three isozymes (*P *<* *0.05). Aroma intensity was also scored significantly lower for tofu produced from Lx12 and Lx123 soybeans, although this difference was not statistically significant for soymilk. On the other hand, sweetness, a positive sensory note, was rated significantly higher in the Lx12 and Lx123 products than the controls for both soymilk and tofu (*P* < 0.05). Fresh pea aroma was also rated higher in the Lx12 and Lx123 products than the controls, although this difference was only significant for tofu. Flavor intensity was rated differently by the taste panels for soymilk and tofu. It was scored lower for the control soymilk (*P *<* *0.05) and higher for the control tofu, although this difference was not statistically different.

## Discussion

Cultural difference and preference, as a result of long‐term or traditional consumption of soy foods, has been reported in the perception of sensory attributes of soy foods, although the results are not clear‐cut (Torres‐Penaranda et al. 1998). The preference for control soymilk from an almost‐native Chinese panel in this study was therefore expected as “traditional” soy users would associate the “beany” flavor with the typical flavor of soy milk. It is possible that consumers of traditional soy foods such as soymilk and tofu may find the products without lipoxygenases too bland due to the absence of the familiar beany flavor and taste. As a result, while soybean varieties lacking lipoxygenases have been developed in countries of traditional soy food consumption such as Korea and China, these varieties have not been widely taken up (K. Lee 2014 and M. Zhang 2013, pers. comm.). For “nontraditional” soy users, the traditional tofu is usually too bland in flavor and mushy in texture to their liking. They may still prefer the seasoned and harder products irrespective of lipoxygenases.

Among the volatile compounds contributing to the beany/rancid/off‐flavor in soy products, hexanal is the major volatile responsible and has been studied most. The lower hexanal level observed in this study in the soymilk and tofu made from the LOX‐free soybeans was consistent with the results from many studies which reported that LOX‐free soybeans produce lower hydroxyperoxide and hexanal levels than those with LOX or lacking the LOX‐1, ‐2, or ‐3 isozyme alone (Hildebrand et al. 1991; Kobayashi et al. 1995; Furuta et al. 1996; King et al. 1998; Ma et al. 2002, 2015). There have also been attempts to link soybean oil profile, lipoxygenase status, and off‐flavor development in soy foods and a direct relationship between the degree of oil polyunsaturation, volatile compounds, hexanal level in particular, and off‐flavor determining parameters has been reported (Furuta et al. 1996; Yuan and Chang 2007; Mandal et al. 2014). The significantly larger increase in the total volatiles in the full‐fat soy meal after 1 week's storage at 37°C was similar to those reported by Kobayashi et al. (1995), although King et al. (1998) observed no difference in hexanal level between control and LOX‐free oils and an increased hexanal level and peroxide values in LOX‐free oil after 2 weeks' storage at 35°C and 50% humidity. The authors attributed these results to the differences in initial fatty acid composition.

The effects of removing lipoxygenases from soybeans on the sensory attributes, in particular on the objectionable beany/grass flavor, of soy products are mixed and seem to be product‐dependent. For example, Torres‐Penaranda et al. (1998) reported significant less cooked beany aroma and flavor in soy milk made from the LOX‐free soybean than that from the normal line, although no difference was observed in tofu in the same study. They also found significant interactions between soybean types and panelist ethnic backgrounds for raw beany aroma and flavor, which was attributed to the low intensity of the raw aroma and flavor making differentiation between soymilk from normal and LOX‐free soybeans difficult. Wilson (1996) also reported that tofu made from LOX‐2 null lines of soybeans were less beany than their respective controls as evaluated by a sensory panel. LOX‐free soybeans, however, showed no effect in oils, breads, and meat patties (King et al. 1998, 2001; Liu et al. 2008). It is possible that the beany flavor was being masked by other stronger sensory notes in these products. In evaluating 70 genotypes including two series of near isogenic lines with or without lipoxygenase isozymes, no effect of lipoxygenase absence was observed on soymilk flavor parameters (Ma et al. 2015). Our results clearly showed that soybeans null in two or three of the major lipoxygenases positively reduced the negative sensory notes including rancidity, bitterness, and aroma intensity and improved the positive sensory notes such as sweetness and fresh pea aroma in soymilk and tofu. Soybeans lacking all three major isomers seemed to have an additive effect on reducing the negative notes in these soy products.

Taken together, our results demonstrated that soybean seeds lacking lipoxygenases can produce whole‐bean‐based soy foods such as soymilk and tofu with improved flavor and aroma which are likely more acceptable to the consumers, particularly “nontraditional” soy users, and perhaps allow their utilization into other products. The soybean lines lacking lipoxygenases will also provide the Australian food industry with additional ingredients that would broaden and increase the utilization of soybeans and soy proteins in a wide range of food applications.

## Conflict of Interest

None declared.
